# Cytokinesis Failure Leading to Chromosome Instability in v-Src-Induced Oncogenesis

**DOI:** 10.3390/ijms18040811

**Published:** 2017-04-12

**Authors:** Yuji Nakayama, Shuhei Soeda, Masayoshi Ikeuchi, Keiko Kakae, Naoto Yamaguchi

**Affiliations:** 1 Department of Biochemistry & Molecular Biology, Kyoto Pharmaceutical University, Kyoto 607-8414, Japan; kd17001@poppy.kyoto-phu.ac.jp (M.I.); ky11084@poppy.kyoto-phu.ac.jp (K.K.); 2 Laboratory of Molecular Cell Biology, Graduate School of Pharmaceutical Sciences, Chiba University, Chiba 260-8675, Japan; soeda05@fc.ritsumei.ac.jp (S.S.); nyama@faculty.chiba-u.jp (N.Y.)

**Keywords:** v-Src, cytokinesis, tetraploidy checkpoint, YAP, chromosome instability

## Abstract

v-Src, an oncogene found in Rous sarcoma virus, is a constitutively active variant of c-Src. Activation of Src is observed frequently in colorectal and breast cancers, and is critical in tumor progression through multiple processes. However, in some experimental conditions, v-Src causes growth suppression and apoptosis. In this review, we highlight recent progress in our understanding of cytokinesis failure and the attenuation of the tetraploidy checkpoint in v-Src-expressing cells. v-Src induces cell cycle changes—such as the accumulation of the 4N cell population—and increases the number of binucleated cells, which is accompanied by an excess number of centrosomes. Time-lapse analysis of v-Src-expressing cells showed that cytokinesis failure is caused by cleavage furrow regression. Microscopic analysis revealed that v-Src induces delocalization of cytokinesis regulators including Aurora B and Mklp1. Tetraploid cell formation is one of the causes of chromosome instability; however, tetraploid cells can be eliminated at the tetraploidy checkpoint. Interestingly, v-Src weakens the tetraploidy checkpoint by inhibiting the nuclear exclusion of the transcription coactivator YAP, which is downstream of the Hippo pathway and its nuclear exclusion is critical in the tetraploidy checkpoint. We also discuss the relationship between v-Src-induced chromosome instability and growth suppression in v-Src-induced oncogenesis.

## 1. Introduction

v-Src is an oncogene that was found in Rous sarcoma virus and its cellular counterpart is c-Src [1,2]. In v-Src, a C-terminal tyrosine residue that is phosphorylated by C-terminal Src kinase (Csk) and is responsible for the closed- and inactive-conformation is lost, resulting in the constitutive activation of v-Src. Src activation is very common in colorectal and breast cancers and is frequently critical in tumor progression through multiple processes, including migration, invasion, anoikis resistance, disruption of cadherin-mediated cell-cell contacts, proliferation, and resistance to apoptosis [1,3]. However, in some experimental conditions, v-Src causes growth suppression [1,4].

Growth promoting factor-induced growth suppression has been reported. The addition of epidermal growth factor (EGF) to serum-free and serum-containing cell culture media results in a marked inhibition of cell proliferation in the A431 human epidermoid carcinoma cell line [5,6,7]. The growth of estrogen-independent breast cancer MDA-MB-468 cells—which overexpress the EGF receptor (EGFR)—is inhibited by EGF; this is accompanied by the increased expression of some genes, including c-myc [8]. Upon treatment with EGF, fibroblastic cells or mammary tumor cells overexpressing EGFR and ErbB2 undergo apoptosis in a p38-dependent manner [9]. Other pathways downstream of the EGFR are also involved in the suppression of cell proliferation. Ras provokes responses to cause cell cycle arrest or apoptosis, although Ras suppresses c-myc-induced apoptosis through the activation of the phosphatidylinositol 3-kinase (PI3K)/Akt pathway [10,11]. Constitutively activated Raf-1, which is a proto-oncogene and a downstream kinase of Ras, increases apoptosis in breast cancer MCF-7 cells [12]. The deregulated expression of c-myc induces apoptosis, although it confers the ability to proliferate in low serum [13,14]. E2F-1 and v-Jun promote both cell cycle progression and apoptosis [15,16]. In addition, the adenovirus protein E1A stimulates transformed focus formation and induces apoptosis, which is inhibited by the E1B protein [17].

Similarly to these growth-promoting factors, v-Src has been reported to cause growth inhibition despite its oncogenic effects. The induced expression of the constitutively active mutant c-SrcY527F has a negative effect on the proliferation of human colorectal cancer HCT116 and SW480 cells in vitro and tumor growth in a xenograft model in vivo [18]. Cell cycle analysis showed the accumulation of cells in the G2 phase of the cell cycle with increased phosphorylation of Tyr15 in Cdk1 and decreased phosphorylation of Ser10 in histone H3 [18]. In Rat-1 fibroblast cells, v-Src does not accelerate the proliferation rate in spite of the decreased expression of the Cdk inhibitor p27 [19]. In carcinoma AA/C1/AB10 cells, an increase of EGFR expression enhances c-Src kinase activity, resulting in increased motility, but not growth stimulation [20]. Furthermore, when Ras and PI3K are inhibited simultaneously, v-Src induces apoptosis in a p53-independent manner [21,22].

Growth suppression and cell death following the hyper-induction of growth factor signaling are thought to be cellular responses to suppress the propagation of unfavorable genes, since activated Ras induces DNA double strand breaks, and other oncogenes, such as myc and E2F1, have similar effects [23,24]. If this is the case, it is thought that growth factor signaling requires further alterations of the gene profiles of primary cells to escape from growth factor-induced growth suppression. One way of altering gene profiles is via changes in gene expression, which is accomplished by gene usage at the transcriptional level, which is observed in v-Src-expressing cells [25]. Another way to alter gene profiles is genetic diversification through chromosome instability. Chromosome instability can be caused by the aberrant segregation of chromosomes during cell division. However, only a few reports have examined the effect of v-Src on genetic stability. In this review we describe our recent findings, including the effect of v-Src on cell proliferation, cytokinesis, and attenuation of the tetraploidy checkpoint, and then discuss the relationship between v-Src-induced chromosome instability and growth suppression in v-Src-induced oncogenesis.

## 2. v-Src Suppresses Cell Proliferation and Induces Tetraploidization

We generated three cell lines derived from mouse fibroblast NIH3T3, human cervix HeLa S3, and human colorectal HCT116 cells—which can induce the expression of v-Src upon treatment with the tetracycline analog doxycycline (Dox)—to observe the effect of v-Src on cell proliferation [4]. When these cells were treated with Dox, v-Src was expressed; phosphorylation of the tyrosine residues in a large number of proteins was enhanced. Cell–cell attachments were weakened, which was accompanied by the loss of E-cadherin staining from the plasma membrane, and the cells were round-shaped. These are the typical features of v-Src expression. v-Src caused growth suppression in these cells, and flow cytometry analysis revealed the alteration of cell cycle progression. While a low concentration of Dox caused the slight accumulation of cells with more than 4N DNA, a high concentration of Dox caused the transient accumulation of 4N cells and then an increase in the sub-G1 population, indicating the induction of apoptosis.

It is noteworthy to mention that the expression level of v-Src in these cells depends on the Dox concentration and the duration of induction. When these cells are treated with a high concentration of Dox, despite induction for only a short period, v-Src is expressed at high levels and induces a variety of effects, including the detachment of the cells from the culture dish. This makes it difficult to perform observations under a microscope. Furthermore, it induces growth suppression and apoptosis as described above. In contrast, a low concentration of Dox induces moderate increases in protein-tyrosine phosphorylation and causes some phenotypes; however, it causes neither detachment of cells nor growth suppression. Thus, these cell lines are thought to be very useful for studying the effect of v-Src at different expression levels without detachment. Furthermore, it is thought that each phenotype caused by v-Src has its own threshold of tyrosine phosphorylation. We chose the concentration of Dox and duration of induction that resulted in less of an effect on both cell proliferation and attachment to the culture dish, but also made it possible to observe the cells under a microscope.

In order to elucidate how 4N cells accumulate following v-Src expression, the cells were treated with the low concentration of Dox for 6 days. Microscopic analysis showed the accumulation of binucleated cells, suggesting that the accumulation of 4N cells is not attributed to G2-arrest. Taken together with the increase in the number of centrosomes, these binucleated cells are generated through cytokinesis failure. Time-lapse analysis of v-Src-expressing cells confirmed that cytokinesis failure is caused by the regression of the cleavage furrow, resulting in the accumulation of 4N cells.

Src family kinases are known to regulate cell division. Upon mitotic entry, Cdk1 induces phosphorylation of the unique domain of Src family kinases, leading to up-regulation of their kinase activities [26,27,28,29,30]. Mitotic entry is inhibited by microinjection of anti-Src antibody into G2 cells [31], and inhibition of Src family kinases blocks mitotic progression in prophase [32]. Cytokinesis is also inhibited by microinjection of anti-Src antibody or Src homology 2 (SH2) domain of Src, or by treatment with the Src inhibitor PP2 [33,34,35]. These results suggest that Src family kinases are required for proper mitotic progression. Each member of the Src family kinases appears to have a distinct role in cell division. Fyn, a member of Src family kinases, participates in the assembly of mitotic spindle microtubules and accelerates mitotic progression [36]. c-Src promotes proper spindle orientation in early prometaphase [37]. Because kinase activities are tightly regulated in a spatiotemporal manner during cell division, we had expected that v-Src—which is aberrantly and constitutively activated—gives rise to uncontrolled cell division including cytokinesis failure.

## 3. v-Src-Induced Cytokinesis Failure Is Caused through Delocalization of Mitotic Regulators

Cytokinesis is the final process that divides cell contents into 2 cells (see reviews, [38,39]). During this process, a signal is transferred from the central spindle to the equatorial cortex. Central spindle formation is regulated by the chromosomal passenger complex (CPC), which consists of Aurora B, INCENP, Survivin, and borealin, but also requires PRC1, KIF4, and Mklp1. Mklp1, a component of the centralspindlin complex, is phosphorylated at Ser708 by Aurora B and is thereby recruited to the spindle midzone. The CPC and centralspindlin complex play roles in contractile ring assembly at the equatorial cortex. After the constriction of the contractile ring, the cell is finally separated into two daughter cells through a final step called abscission, in which ESCRT-III proteins catalyze the scission of membrane necks. Abscission timing is regulated by Plk1 and Aurora B kinases, and Aurora B prevents premature abscission if chromosome bridges are present in the intracellular bridges.

**Figure 1:**
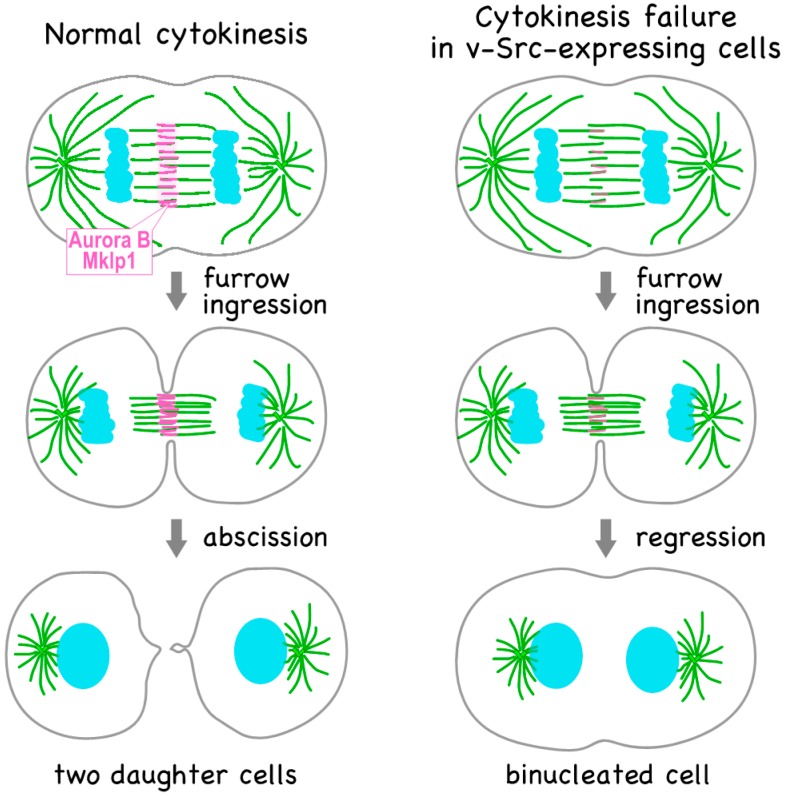
v-Src induces cytokinesis failure. The chromosomal passenger complex (CPC) and centralspindlin complex are localized at the spindle midzone; however, v-Src causes delocalization of the components of these complexes. As a result, cytokinesis fails due to regression of the cleavage furrow, resulting in the formation of binucleated cell.

In order to explore the mechanism underlying v-Src-induced cytokinesis failure, we examined the localization of cytokinesis regulators and found the delocalization of the kinesin motor proteins Mklp1 and Mklp2, and the components of the CPC—Aurora B kinase and INCENP—from the anaphase midzone (Figure 1) [4]. Cleavage furrow ingression requires the localization of Mklp1; v-Src-induced cytokinesis failure is caused by Mklp1 delocalization. Given that v-Src downregulates cell adhesion [4,40], and that cell adhesion interacts with cytokinesis [41,42], v-Src-induced cytokinesis failure would be caused by detachment of v-Src-expressing cells. We examined suspension cultures of HeLa S3 cells to exclude the possibility that the detachment of v-Src-expressing cells is involved in v-Src-induced cytokinesis failure. Similar to cells attached to culture dishes, Aurora B was localized to the anaphase midzone in the absence of v-Src expression and delocalized upon v-Src expression in suspension-cultured HeLa S3 cells, excluding the possibility that cytokinesis failure is caused by the loss of adhesion of v-Src-expressing cells.

How are cytokinesis regulators delocalized by v-Src? A plausible hypothesis is the inhibition of the kinesin-like motor protein Mklp2, which is responsible for the relocation of the CPC from the centromeres to the anaphase spindle midzone [43]. Knockdown of Mklp2 causes failure of abscission, generating binucleated cells [44]. In the absence of Mklp2, Mklp1 phosphorylation at Ser708, which is required for the recruitment of Mklp1 to the midzone, is lost [45]. This phosphorylation is catalyzed by Aurora B, and when Mklp2 is knocked down, Aurora B cannot relocate to the anaphase spindle midzone and remains with segregating chromosomes at anaphase [43]. Thus, Aurora B cannot phosphorylate Mklp1 at Ser708, resulting in the delocalization of Mklp1 and thereby causing cytokinesis failure. However, this hypothesis is unlikely to be true, since Aurora B never co-localizes with segregating chromosomes upon v-Src expression.

Interestingly, the delocalization of Aurora B was observed after a 15-min incubation of v-Src-expressing cells with the Aurora B inhibitor ZM447439 [4]. This means that the kinase activity of Aurora B is required to maintain its localization to the anaphase spindle midzone, in agreement with a previous report [46]. When the cell cycle was synchronized by treatment with nocodazole, the auto-phosphorylation of Aurora B was not different between v-Src-expressing and v-Src-non-expressing cells. However, kinase activity should be evaluated in anaphase cells and not in nocodazole-synchronized cells. Considering that the duration of anaphase is generally short, it seems to be difficult to examine protein modifications at anaphase by western blotting because of the difficulty of synchronizing the cell cycle at anaphase. However, we developed a method to synchronize cells at anaphase by using a low concentration of nocodazole and blebbistatin and optimizing the period of treatment with the reagents, by which approximately 40% of cells were synchronized at anaphase [47]; nevertheless, it is still difficult to synchronize the cell cycle in v-Src-expressing cells due to the changes in the cell cycle [4,18] and partial disruption of the spindle assembly checkpoint [4]. Thus, after optimization of the protocol for synchronizing v-Src-expressing cells, further studies including a search for v-Src substrates would help to understand the mechanism underlying the v-Src-induced delocalization of cytokinesis regulators.

## 4. v-Src Attenuates the Tetraploidy Checkpoint

Although tetraploidy is one of the causes of chromosome instability through the increase in the number of centrosomes [48,49] and promotes tumorigenesis in p53-null cells [50], tetraploid cells can be removed by a mechanism called the tetraploidy checkpoint [51,52,53]. However, we observed a time-dependent increase in the number of binucleated cells upon the induced expression of v-Src in human colon carcinoma HCT116 and mouse fibroblast NIH3T3 cells, which is accompanied by the accumulation of the 4N cell population on flow cytometry analysis. Furthermore, we also observed an increase in the number of cells having multipolar spindles with excess centrosomes. These results suggest that v-Src causes chromosome instability through cytokinesis failure and the resulting tetraploidization. However, given that cell cycle progression is prevented in tetraploid cells by the tetraploidy checkpoint, tetraploid cells generated by v-Src expression should be arrested at the G1 phase with 4N DNA content. Thus, the increase in the number of cells with multipolar spindles raised the possibility that the tetraploidy checkpoint is attenuated in v-Src-expressing cells.

**Figure 2:**
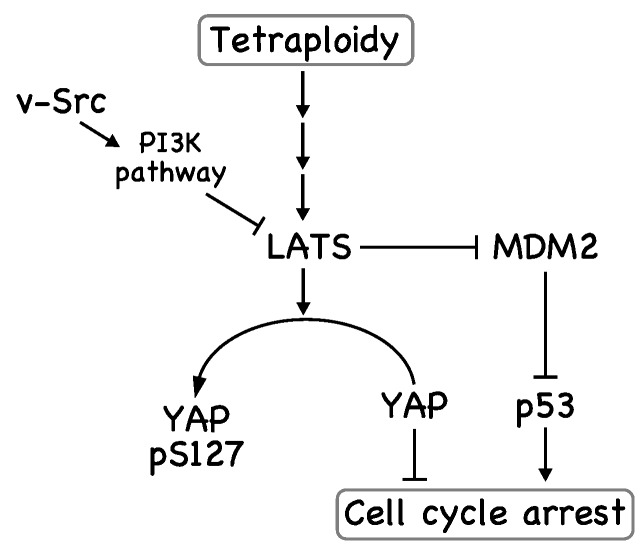
v-Src weakens the tetraploidy checkpoint. LATS kinases, which are activated in tetraploid cells, phosphorylate YAP, leading to exclusion of YAP from the nucleus. In addition, LATS activation results in stabilization of p53 through inhibition of MDM2. As a result, tetraploid cells are removed at this tetraploidy checkpoint. v-Src inhibits YAP phosphorylation by inhibiting LATS, possibly through activation of the PI3K pathway.

The Hippo pathway is reportedly activated in tetraploid cells; LATS kinases are activated, resulting in the inhibition of the transcriptional regulators YAP and TAZ and the stabilization of p53 (Figure 2) [53]. In this case, YAP is phosphorylated by LATS2 and excluded from the nucleus. In addition, LATS2 binds to and inhibits MDM2—an E3 ubiquitin ligase for p53—leading to p53 stabilization [54]. We observed that the subcellular localization of YAP in NIH3T3 cells depended on cell density, suggesting that its subcellular localization is downstream of the Hippo pathway in NIH3T3 cells [4]. This subcellular localization was found to be partially regulated by Src kinase activity, since Src inhibition by the Src inhibitor PP2 led to the cytoplasmic localization of YAP, and v-Src expression led to the nuclear localization of YAP. When NIH3T3 cells were treated with cytochalasin B or the Plk1 inhibitor BI2536, the number of binucleated cells was increased through cytokinesis failure. In these binucleated cells, the cytoplasmic localization of YAP was promoted, suggesting activation of the Hippo pathway. However, v-Src expression led to the nuclear localization of YAP in most of the binucleated NIH3T3 cells. In agreement with these subcellular localizations, v-Src inhibited the phosphorylation of LATS kinases and YAP, indicating that v-Src may inhibit LATS kinases and thereby inhibits YAP phosphorylation, although the v-Src substrate responsible for this phenotype has not been determined. Therefore, v-Src may weaken the tetraploidy checkpoint by attenuating the Hippo pathway.

One possible mechanism underlying the v-Src-induced suppression of the Hippo pathway could be phosphorylation of YAP by v-Src. YAP was isolated initially as a Yes-associated protein, where YAP binds to the Src homology 3 (SH3) domain of Yes, a member of the Src-family kinases [55], in a manner that depends on Yes kinase activity [56]. YAP is phosphorylated at Tyr357 by Yes [57], resulting in the nuclear localization of YAP [58]. Abl tyrosine kinase also phosphorylates YAP at this site, leading to the stabilization of YAP [59]. However, we never observed an increase in the expression level of YAP upon v-Src expression. Thus, direct phosphorylation of YAP at Tyr357 by v-Src may not be responsible for the v-Src-induced nuclear localization of YAP in our experimental conditions. Another possibility is activation of PI3K by v-Src. The FAK-Src-PI3K pathway reportedly inhibits the Hippo pathway, leading to a decrease in YAP phosphorylation at Ser127 [60]. Since v-Src is also known as an activator of PI3K, v-Src may activate PI3K and reduce the activity of LATS kinases.

The tumor suppressor p53 also plays an important role in the suppression of the cell cycle in tetraploid cells (Figure 2) [54]. We also observed a decrease in p53 levels in v-Src-expressing cells [40]. p53 degradation is regulated by the E3 ubiquitin ligase MDM2 [61,62,63,64], which is inactivated by LATS2 [54]. Because we observed the v-Src-induced reduction of YAP phosphorylation at Ser127, a substrate of LATS kinases, the inactivation of LATS kinases could be the underlying mechanism; namely, inactivated LATS kinases cannot prevent MDM2-mediated p53 degradation, leading to a decrease in p53 levels. Src also reduces p53 levels in a different way; Src inactivates the phosphatase PP2A by phosphorylating Tyr307, leading to MDM2-mediated p53 degradation through an increase in MDM2 phosphorylation at Ser166, which enhances its interaction with p53 [65]. Furthermore, strong growth signals can overcome cell cycle arrest in tetraploid cells [53]. v-Src stimulates growth signals, such as ERK and Akt, contributing to the silencing of the tetraploidy checkpoint. Taken together, v-Src may weaken the tetraploidy checkpoint through multiple pathways.

## 5. v-Src Can Induce Chromosome Instability, Generating Genetic Diversity

One of the causes of chromosome instability is the formation of multipolar spindles [66]. An excess number of centrosomes lead to the asymmetrical segregation of chromosomes through multipolar spindle formation and the following multipolar anaphase. However, an excess number of centrosomes tend to form bipolar spindles by centrosome clustering; that is, centrosomes gather into two poles after the formation of multipolar spindle intermediates [48]. These plausible bipolar spindles function incorrectly, since lagging chromosomes are observed frequently during anaphase [48]. For bipolar spindles with an excess number of centrosomes, merotelic kinetochore-microtubule attachment errors occur easily, resulting in lagging chromosomes [48,66,67,68]. Thus, dysregulation of the number of centrosomes results in chromosome instability.

v-Src induces tetraploid cells through cytokinesis failure. Tetraploid cells have an excess number of centrosomes, which can form multipolar spindles. Even though pole clustering prevents cells from forming multipolar spindles, transient multipolar spindle intermediates favor the formation of merotelic attachments [48]. This can lead to the premature onset of anaphase with lagging chromosomes, resulting in the formation of a micronucleus [69]. Chromosomes in micronuclei undergo complex and localized genomic rearrangements through a process called chromothripsis, in which chromosomes are fragmented and re-ligated by non-homologous end joining [69,70,71]. Thus, v-Src-induced cytokinesis failure is a trigger for v-Src-induced genetic diversification.

We reported that non-membrane-bound Src-family kinases induce formation of chromosome bridges [72], and that v-Src also induces them in a caffeine-dependent manner through induction of DNA damage [73]. Chromosome bridges result in the generation of daughter cells with different numbers of chromosomes and thus generate genetic diversity. Furthermore, cytokinesis in cells with chromosome bridges sometimes fails if the chromosome bridges are not resolved before abscission. Chromosome bridges activate the abscission checkpoint that prevents cells in the abscission phase from furrow regression [74]. However, given that the abscission checkpoint requires Aurora B kinase activity [75,76,77], v-Src may disrupt the abscission checkpoint and induce furrow regression of cells with chromosome bridges through delocalization of Aurora B from the midbody. Thus, v-Src can cause cytokinesis failure more frequently in chromosome bridge-containing cells than in cells without chromosome bridges.

Aurora B regulates the appropriate binding of microtubules and kinetochores; monotelic and syntelic attachments are corrected at the spindle assembly checkpoint, which requires Aurora B kinase activity [78,79]. In addition, merotelic attachments are also corrected by a mechanism dependent on Aurora B and MCAK [80,81]. If the delocalization of Aurora B that was observed in v-Src-expressing anaphase cells [4] is caused before anaphase onset, it may give rise to erroneous chromosome segregation through inappropriate microtubule-kinetochore attachments. This can lead to further cytokinesis failure.

## 6. Conclusions and Perspectives

**Figure 3:**
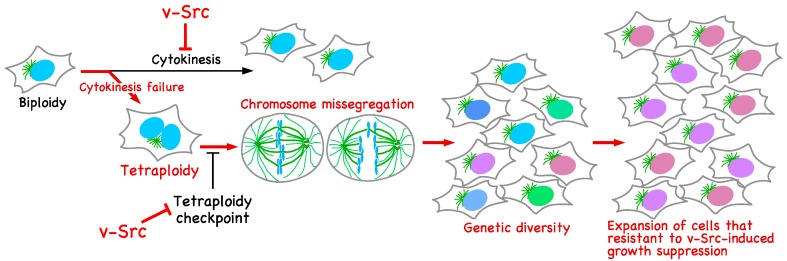
Genetic diversification in v-Src-expressing cells. v-Src induces tetraploidization through cytokinesis failure. The activation of the tetraploidy checkpoint in tetraploid cells is suppressed by v-Src. Tetraploid cells give rise to chromosome mis-segregation, leading to genetic diversification. v-Src causes growth suppression; however, among cells with broad genetic diversity, cells resistant to v-Src-induced growth suppression can evolve and continue to proliferate.

Genetic alteration through chromosome instability causes growth suppression. For example, aneuploid yeast cells show proliferative disadvantages, such as defects in cell cycle progression [82]. Mis-segregation of chromosomes in human diploid cells delays cell cycle progression, and the p53 pathway plays an important role in limiting the proliferation of aneuploid human cells [83]. Although the overexpression of Mad2, which is an essential component of the spindle assembly checkpoint, together with Kras^G12D^ delays Kras-driven tumor initiation, Mad2 overexpression facilitates oncogene-independent outgrowth and tumorigenesis in mice through chromosome instability [84,85,86]. This suggests that chromosome instability-driven genetic diversity can result in subclones and promote adaption for a strong selective pressure, such as the loss of growth signals by the withdrawal of oncogene and cell cycle arrest at checkpoints [87,88,89]. Although it has long been known that v-Src has oncogenic potential and accelerates malignant progression [1], it also causes growth suppression, as we observed in some cell lines [4]. We expect that the generation of genetic diversity is required for-v-Src-expressing cells in order to overcome v-Src-induced growth suppression. Here, we propose a model for v-Src-induced oncogenesis in addition to the canonical roles of v-Src (Figure 3). During v-Src-induced oncogenesis, v-Src induces tetraploidization through cytokinesis failure. Then, the activation of the tetraploidy checkpoint in tetraploid cells is suppressed by v-Src, leading to genetic diversification through chromosome instability. Among cells with broad genetic diversity, clones that adapt to growth-suppressive circumstances evolve and continue to proliferate or acquire the capacity to metastasize.

Indeed, increases in Src activity are frequently observed in colon and breast cancers. In addition to the roles of Src activity in tumor development and malignancy through a variety of processes, we have shown that an increase in Src activity has the potential to affect genetic stability. We observed that overexpression of c-Src induces only slight increases in the number of binucleated cells [4], suggesting that further activation may be required for induction of cytokinesis failure. As our work was performed by using v-Src as a model for a constitutively active version of Src, further study is needed to determine the involvement of c-Src that is activated by a mutation in the C-terminal inhibitory tyrosine residue or by suppression of Csk activity. Csk is recruited to the vicinity of active Src in plasma membrane lipid rafts by binding to the transmembrane adaptor protein PAG/Cbp [90,91]. Thus, PAG/Cbp act as a negative regulator of Src. Src stimulates the PI3K and ERK pathways, leading to histone modifications that suppress PAG/Cbp synthesis at the transcriptional level, indicating the presence of a positive-feedback loop in oncogenic signaling [92]. It would be interesting to examine whether knockdown of PAG/Cbp expression causes chromosome instability.

v-Src has the potential to induce epithelial-mesenchymal transition (EMT), which contributes to cancer progression [93,94,95]. Transcription factors including Snail, zinc-finger E-box-binding (ZEB) and basic helix-loop-helix (bHLH) drive EMT through repression of the epithelial marker genes and activation of the mesenchymal phenotype-associated genes [95]. The MEK/ERK pathway participates in EMT by increasing the expression of EMT transcription factors, and is also linked with the downregulation of E-cadherin in a manner independent of EMT transcription factors; downregulation of E-cadherin is a hallmark of EMT, which induces disassembly of adherens junctions. The MEK/ERK pathway causes overexpression of Cdc6, leading to repression of E-cadherin transcription [96]. Adherens junction maintenance requires the Rac activator Tiam1. During Src-induced disassembly of adherens junctions, Tiam1 is phosphorylated and degraded through activation of the MEK/ERK pathway [97]. As previously mentioned, v-Src stimulates the MEK/ERK pathway, suggesting that the MEK/ERK pathway is involved in the v-Src-induced EMT in both EMT transcription factor-dependent and -independent manners. As the effects of v-Src-induced genetic diversity on EMT have not yet been explored, further study is required to determine how chromosome instability caused by v-Src relates to cancer malignancy, including metastasis, in a mouse model.

## Abbreviations

Csk	C-terminal Src kinase
EGF	epidermal growth factor
CPC	chromosomal passenger complex
SH2	Src homology 2
SH3	Src homology 3
